# A microfluidics-based method for isolation and visualization of cells based on receptor-ligand interactions

**DOI:** 10.1371/journal.pone.0274601

**Published:** 2022-10-06

**Authors:** Long Dao, Qingnan Zhao, Jiemiao Hu, Xueqing Xia, Qing Yang, Shulin Li

**Affiliations:** Department of Pediatrics, The University of Texas MD Anderson Cancer Center, Houston, Texas, United States of America; University of Pisa, ITALY

## Abstract

Receptor-ligand binding has been analyzed at the protein level using isothermal titration calorimetry and surface plasmon resonance and at the cellular level using interaction-associated downstream gene induction/suppression. However, no currently available technique can characterize this interaction directly through visualization. In addition, all available assays require a large pool of cells; no assay capable of analyzing receptor-ligand interactions at the single-cell level is publicly available. Here, we describe a new microfluidic chip–based technique for analyzing and visualizing these interactions at the single-cell level. First, a protein is immobilized on a glass slide and a low-flow-rate pump is used to isolate cells that express receptors that bind to the immobilized ligand. Specifically, we demonstrate the efficacy of this technique by immobilizing biotin-conjugated FGL2 on an avidin-coated slide chip and passing a mixture of GFP-labeled wild-type T cells and RFP-labeled FcγRIIB-knockout T cells through the chip. Using automated scanning and counting, we found a large number of GFP+ T cells with binding activity but significantly fewer RFP+ FcγRIIB-knockout T cells. We further isolated T cells expressing a membrane-anchored, tumor-targeted IL-12 based on the receptor’s affinity to vimentin to confirm the versatility of our technique. This protocol allows researchers to isolate receptor-expressing cells in about 4 hours for further downstream processing.

## Introduction

Receptor-ligand interactions are a crucial class of protein-protein interactions. At the cellular level, these interactions are responsible for a great variety of processes, including immune activation, metabolic changes, and neurotransmission [1–3]. As such, the measurement and visualization of these interactions is tremendously useful in the study of various cell types. Receptor-ligand interactions are of a noncovalent nature but are not as strong or as specific as antibody-antigen interactions [4]. Thus, currently, studies of ligand-receptor interactions require large populations of cells and cannot focus on the single-cell level. Although the ability to isolate cells based on ligand-receptor interactions would prove valuable for studies regarding binding preferences and signaling mechanisms at the single-cell level, no such method currently exists. Current methods can isolate the receptor and ligand proteins, but not the cells on which these macromolecules are expressed [5]. Moreover, as visualization would help to validate the detected interactions, the technique could be further strengthened by adding a visual component.

Methods used to investigate receptor-ligand interactions typically employ immobilized receptors that are titrated with fluorophore- or radiolabeled ligands [6]. Similarly, surface plasmon resonance (SPR) also involves the titration of receptors, albeit with unlabeled ligands. In SPR, the ligand binds to the receptor on a planar metal, changing the refractive index of the material; this change is used to determine the binding affinity of the ligand and receptor [7]. However, fluorophore and radiolabeled ligand titration and SPR have to be performed using purified proteins or cell lysates. Some groups have reported success in immobilizing whole cells prior to binding assays with the labeled binding and SPR techniques. However, these assays are more appropriate for studies in which the cells themselves are not the subject of the study, but rather the means by which surface receptors are expressed [8]. Another caveat regarding these whole cell techniques is that the cells must be of a single phenotypic signature. This requirement can cause complications when using cells generated from animal models, as isolation and purification of cells may not lead to a completely uniform cell type. Additionally, cell-surface receptor expression may change during isolation and purification. Like SPR, isothermal titration calorimetry (ITC) uses unlabeled ligands and receptors; a ligand and its receptor are titrated, and the change in heat is measured. However, a whole-cell approach to ITC is unfeasible. Thus, to our knowledge, no published works have described the isolation of cells on the basis of ligand-receptor interactions. Furthermore, the available techniques do not support direct visualization by either classical or current imaging systems at the single-cell level. Such visualization would provide definitive validation of the detected interaction.

Here, we demonstrate a technique with which to isolate and visualize cells based on FcγRIIB receptor affinity to FGL2 ligand [9]. Our method utilizes a ligand-coated slide chip and a microfluidic pump that slowly moves cells through microfluidic channels on the slide chip. Cells that do not interact with the bound ligand are moved into a waste container, leaving only the cells of interest. Following this, the cells can be conventionally stained and imaged for further analysis.

## Materials and methods

The protocol described in this peer-reviewed article is published on protocols.io, dx.doi.org/10.17504/protocols.io.cf9etr3e and is included for printing as supporting information file 1 with this article.

### Equipment

#### Hardware

Cytoquest Platform (Abnova, cat. no. M0014-04)
SCx^™^ Spiral Chamber (Abnova, cat. no. U0314)
Keyence Microscope (Keyence, model no. BZX-710)
CytoChipNano CR (Abnova, cat. No. U0096)

#### Software

CytoQuest_CR_3.2 (Abnova)
BZ-X Analyzer Software (Keyence)

#### Mice

FcγRIIB-KO (Taconnic, model no. 580)
C57/BL6 mice (Jackson, cat. No. 000664)

#### Reagents

MojoSort Mouse CD3 T cell Isolation Kit (BioLegend, cat. no. 480023)
CellTracker Green BODIPY dye (Thermo Fisher Scientific, cat. no. C2102)
CellTracker Red CMTPX dye (Thermo Fisher Scientific, cat. no. C34552)
Streptavidin (Sigma, cat. no. 189730)

### Procedure

**Fig 1:**
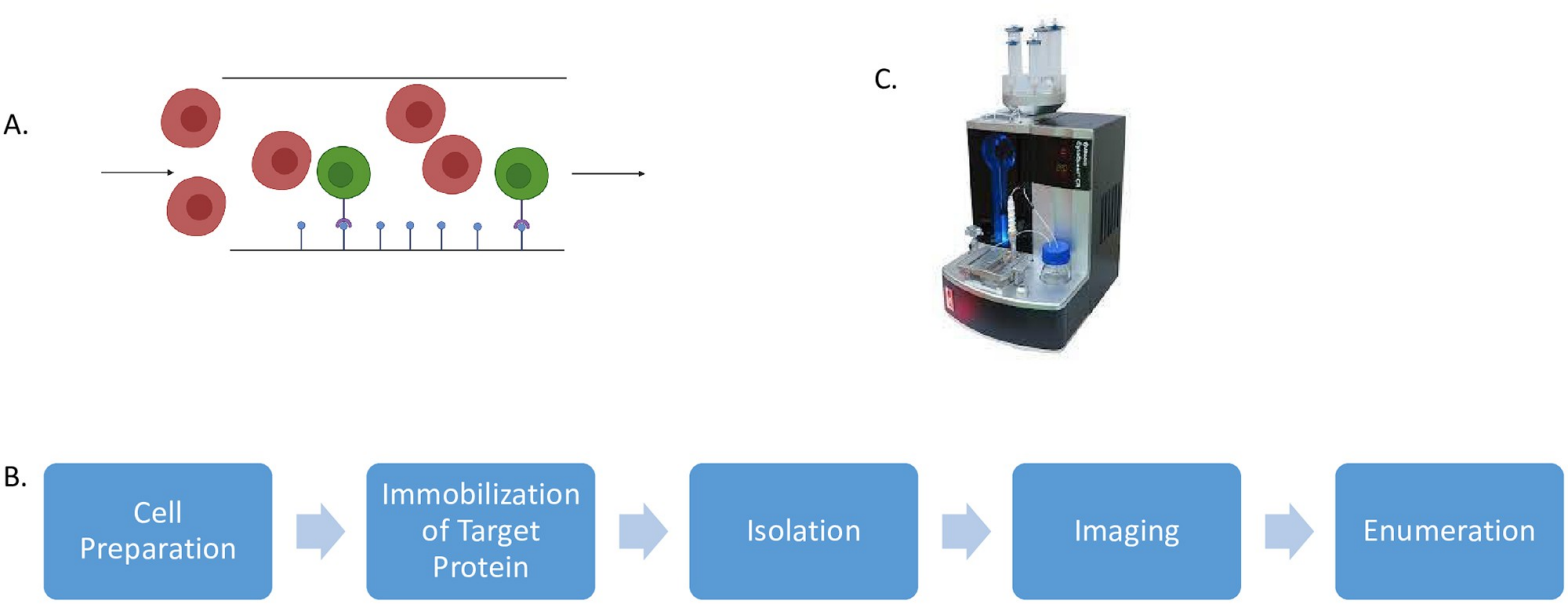
Cytoquest schematic and workflow. (A) Cellular level diagram of ligand coated slide chamber coated microfluidic slide chamber. Green cells express the appropriate receptor to the ligand and are captured onto the chamber. (B) Workflow diagram. (C) Abnova Cytoquest.

This protocol has five main steps: generation and labeling of cells, immobilization of target proteins, isolation of cells, automated imaging, and cell enumeration. The basis of the technique is illustrated in Fig 1A, and the resulting workflow is shown in Fig 1B.

1. Generation and labeling of cells
  Extract and homogenize the spleens of FcγRIIB-KO (Taconic, model no. 580) and C57/BL6 mice (Jackson Labs, cat. no. 000664) with a 40-μm mesh filter. T cells were then isolated from the homogenized cells by using a MojoSort Mouse CD3 T cell Isolation Kit (BioLegend, cat. no. 480023). Label T cells from WT mice with CellTracker Green BODIPY dye (Thermo Fisher Scientific, cat. no. C2102), and T cells from FcγRIIB-KO mice with CellTracker Red CMTPX dye (Thermo Fisher Scientific, cat. no. C34552).
2. Immobilization of target protein
  Immobilize FGL2 on the microfluidic chip by covering a polydimethylsiloxane-coated microfluidic chip with 1 mg/mL streptavidin (Sigma, cat. no. 189730) for 1 hour at room temperature. Then, coat the chip with 32 μg of biotin-conjugated FGL2 suspended in 100 μL of phosphate-buffered saline (PBS) for 1 hour at room temperature.
3. Isolation
  Mix 1.5 × 10^6^ FcγRIIB-KO T cells (red) and 1.5 × 10^6^ WT T cells (green) and resuspend in 100 μL of T Cell media. Connect a spiral chamber containing microfluidic tubing to the Abnova CytoQuest microfluidic pump and prime for capture of WT T cells by washing with PBS and water. Attach the chamber to the pumpTemporarily disconnect the chamber from the pump and place the end tubing into an Eppendorf tube containing the T-cell mixture. The pump will draw the T cells into the chamber tubing and then passed the cell mixture through the slide chip. Only cells that display strong binding to the recombinant FGL2 protein will remain on the microfluidic chip. Cells that do not bind strongly will pass through the microfluidic chip into a waste container.
4. Visualization
  Image the microfluidic chip with an automated Keyence microscope (www.keyence.com, model no. BZX-710) at 20× magnification. Assign individual set points were and image for fluorescein isothiocyanate and phycoerythrin channels. The duration of this process was between 3 and 4 hours.
5. Enumeration
  Use the BZX-800 analysis software (Keyence) to count the cells on the resulting images. Count individual images were ‘stitch mode’ in groups of 400 until every image is counted.

#### Timing

Step 1, generation and labelling of cells 1–1.5 hours
Step 2, Immobilization of target protein, 2–2.5 hours
Step 3, Isolation, 1.5–2.5 hours
Step 4 Visualization, 3–4 hours
Step 5 Enumeration, 4–8 hours

## Results and discussion

### Limitations

Although this technique is universally adaptable to different cell types, it is limited to use with single-cell suspensions only. Thus, tissues must be transformed into single-cell suspensions before using this technique. The dissociation process can produce changes in gene expression and, therefore, protein expression, which may cause misleading results [10]. Optimization of tissue dissociation may prove time consuming.

Additionally, this method can isolate cells only based on surface protein expression, not expression of cytoplasmic or nuclear proteins. In cell lineage studies, this may prove a hindrance, as transcription factors must often be identified to distinguish cell types [11]. Future research may be targeted at isolation of cell types based on expression of these proteins. Furthermore, if two cell types of interest do not have sufficiently different expression levels of a surface protein, this technique cannot be used. However, even if both cell types express the same surface receptor, they can both be stained and examined for expression of other proteins.

Unlike some other bioanalytical methods, this technique does not quantify binding affinity and is therefore inappropriate for biophysical studies.

### Comparison with other approaches

#### Cellular isolation

Fluorescence-activated cell sorting (FACS) and magnetic-activated cell sorting (MACS) are the most direct comparisons for the cellular isolation aspect of our technique. In the FACS process, cells are labeled with fluorophore-tagged antibodies and processed through a flow cytometer, which diverts cells of interest into a chamber on the basis of the user’s gating strategy. In the MACS process, cells are labeled with a magnetic bead–tagged antibody and processed through a strong magnetic field, which leaves behind cells expressing the protein of interest. The crucial difference between our technique and FACS and MACS is that our technique is based on receptor-ligand interactions rather than antibody-antigen interactions.

In comparison to FACS, our technique offers several benefits. First, it directly compares the binding preferences of two different cell types. Additionally, the cells isolated using our technique are only bound to the antibody on the chip, whereas cells isolated using FACS can be bound to several antibodies.

Our technique also offers several benefits over MACS. MACS typically involves more manipulation of cells than our technique does [12]. During this manipulation, cells may be lost. While this loss is of no consequence for common cell types, it can be catastrophic for studies requiring examination of rare cells. Our technique, however, requires very little manipulation of the cells and is therefore more suitable for analyses of rare cell types. Additionally, our technique does not involve metal tagging, which may present an issue for techniques that substitute metal isotopes for fluorophores, such as imaging mass cytometry and time-of-flight mass cytometry.

Our methodology is more capable of isolating and visualizing rare cells than is either FACS or MACS because it was originally intended to capture CTCs, which are rare. In contrast, MACS and FACS both require large amounts of cells for isolation and visualization. Additionally, our technique can visualize the interaction between the receptor and the ligand, which may provide information about the manner in which the ligand and receptor interact that may not be otherwise available.

The main disadvantage of our technique in comparison to MACS and FACS is that it isolates cells onto a slide rather than into a tube [13]. If the cells are intended to be used for microscopy or other techniques for which slides are appropriate, this is inconsequential. Another shortcoming of our technique is that it is more expensive than FACS or MACS because both the slide chip and the spiral chamber are single-use consumables.

#### Binding affinity

ITC is the most directly comparable method for comparing the binding preferences of two different cell types. In this process, molecules are titrated against other molecules in a sample cell enclosed in an adiabatic jacket [14]. A reference cell containing a buffer is also enclosed in the jacket. During the titration process, the change in heat is measured and used to determine binding affinity, change in enthalpy, and binding stoichiometry.

Our technique has several advantages over ITC. First, it requires far less manipulation of cells. Proteins must maintain their folded structure to properly bind to their targets, and keeping a membrane protein in its native folded state can often be challenging [15, 16]. This challenge can be compounded by the high concentrations of proteins required for ITC analysis. Because our technique keeps proteins intact on the cell membrane, it can bypass challenges associated with protein folding, solubility and, stability [17]. Additionally, ITC often requires large amounts of membrane proteins to run assays at various concentrations in triplicate. Thus, proteins to be studied using ITC must be expressed and purified in large quantities [17]. Finally, as our technique keeps the cells expressing the protein of interest intact, the cells can be stained for other proteins.

The main shortcoming of our technique in comparison with ITC is that our technique does not give binding affinities, changes in enthalpy, or binding stoichiometry. Although our technique reveals whether a certain cell type preferentially binds to a given protein, it does so only as a ratio of one cell type to another. Additionally, our technique is far less sensitive than ITC. ITC can detect changes in heat at ranges from approximately 1 to 50 μM, whereas our technique requires 400 μM of protein for capture. Another weakness of our technique is that it requires more single-use disposable equipment, which may result in a higher long-term cost.

Ligand binding assays are another source of comparisons among binding techniques. All of these techniques depend on the titration of immobilized receptors with a ligand [18]. Our technique imparts several advantages over these assays. First, our technique is safer and more cost effective than radiolabeled ligand binding assays and works in the opposite way. In labeled ligand binding assays, the receptors are immobilized to a surface and the ligand is used to titrate the cells. In our method, the ligands are immobilized and receptors are presented on the cell surface are used to titrate the ligands. By inverting what would be the mobile and stationary phases, we are able to capture and visualize the cells that express the receptors. A second advantage of our technique in comparison with both labeled ligand binding assays and SPR is that our technique can determine the binding preferences of two cell types in a single assay. SPR and labeled ligand binding assays would require two experiments to compare the binding preferences of two cell types. Third, our technique is more user friendly than SPR and requires less training time [19]. Finally, visualization of cellular receptor-ligand interactions is another crucial advantage of our method over ITC and SPR. Visualization allows investigators to confirm the receptor-ligand interaction. Because of the sensitivity of ITC, individual receptors and ligands must be used in place of whole cells. Thus, the interaction at the cellular level cannot be visualized. Similarly, although there are publications using whole cells for SPR, there have been no reports of microscopy following SPR [8].

Again, the main disadvantage of our technique its inability to reveal the binding affinity of the receptor and ligand. Our technique also has some of the same drawbacks of fluorescent-ligand binding assays, that is, spectral overlap issues. In addition to providing binding affinity, SPR and labeled ligand binding assays provide binding kinetics, whereas our assay cannot [19]. Labeled ligand binding assays also require less specialized instrumentation than our technique does. Comparisons between these methods are outlined in Table 1.

**Table 1:** Comparison of techniques for the analysis of cell-surface receptor-ligand interactions.

Technique	Advantages	Limitations
Microfluidic chip-based isolation and visualization	Can visualize cell-surface ligand-receptor interactions	Tissue dissociation for single cell suspension may change gene expression
	Cellular isolation is possible based on receptor-ligand interactions	Limited to cell surface proteins
		Does not quantify binding affinity
	Can give qualitative insight into receptor-ligand interactions	Isolation is limited to slide chip
	Equipment can be costly	Low loss of rare cells
Fluorescence-activated cell sorting	Cells can be isolated for multiple downstream uses	Cells are not visualized
	Cells can be isolated based on multiple proteins	
Magnetic-activated cell sorting	Cells can be isolated for multiple downstream uses	Cells are not visualized
	Cells can be isolated based on multiple proteins	
	May require optimization for rare cell types	
Isothermal titration calorimetry	Quantifies binding affinity, change in enthalpy and binding stoichiometry	Cells are not visualized
		Protein stability may affect results
	Highly sensitive	Can require large amounts of protein
		Only one protein can be examined at a time
Radiolabeled ligand binding assay	Quantifies binding affinity and kinetics	Cells are not visualized
Surface plasmon resonance	Quantifies binding affinity and kinetics	Cells are not visualized
	Only one interaction can be assessed per assay	

### Applications of the method

**Fig 2:**
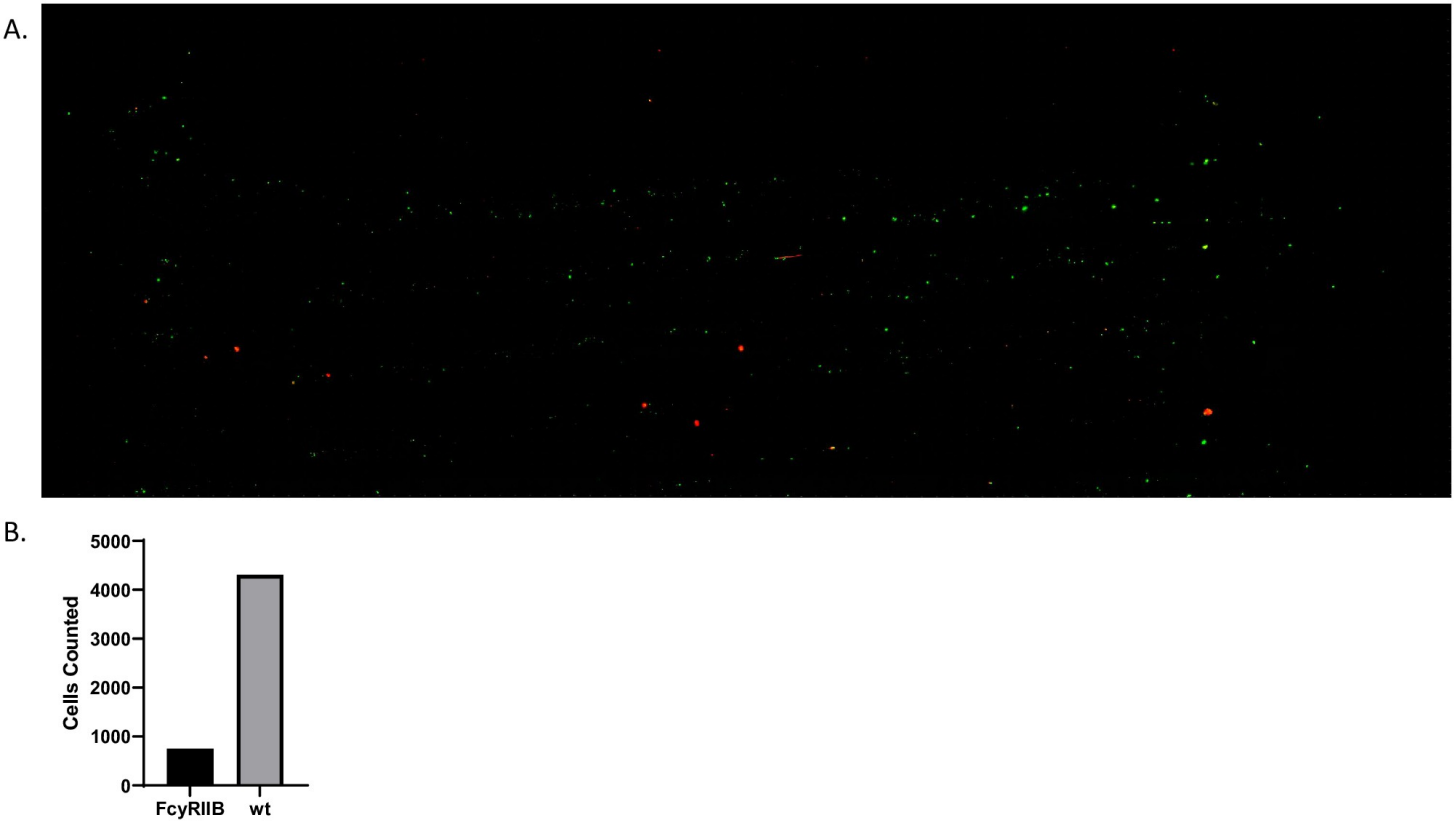
The Cytoquest is capable of separating wt T cells from FcγRIIB based on FGL2 expression. (A) Fluorescent microscopic imaging of FGL-2 coated slide chip after being loaded and washed with FcγRIIB KO T Cells (red) and wt T Cells (green) (B) Quantification of cells.

This method can be used to definitively validate the interaction between a ligand and its receptors at a high-resolution cellular level. FcγRIIB was known to be a receptor of the FGL2 ligand, but the only evidence available was from gene expression assays [20]. The method described here definitively confirmed that FcγRIIB-WT T cells (green) can bind the FGL2-coated chip, whereas FcγRIIB-KO T cells (red) cannot (Fig 2A).

**Fig 3:**
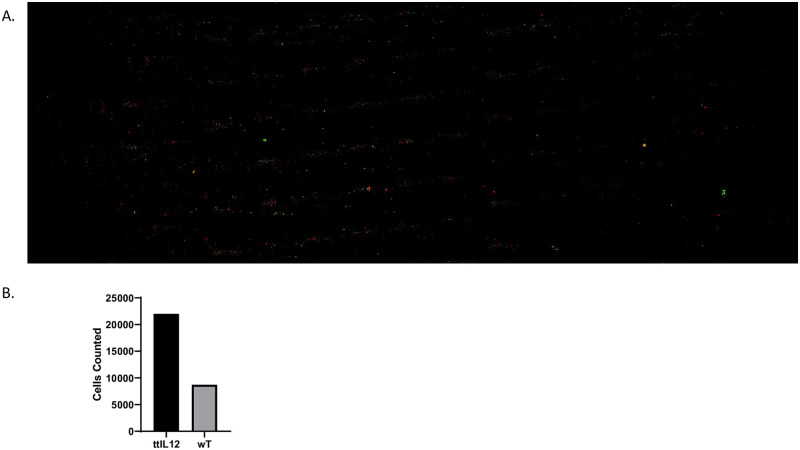
The Cytoquest is capable of isolating ttIL12 T cells from wt T cells based on Vimentin expression. (A) Fluorescent microscopic imaging of vimentin coated slide chip after being loaded and washed with ttIL12 T Cells (red) and wt T Cells (green) (B) Quantification of cells.

To validate this method, we used an independent system employing vimentin-binding ATTIL-12 T cells (green) and control T cells (red). As can be seen in Fig 3A, the cells are predominantly green. Compared to the interaction of FGL2 and its cognate receptor-positive T cells, control-T cells also heavily bound vimentin (22,011 ATTIL12-T cells vs. 8725 control-T cells; Fig 3B), suggesting that vimentin may bind other cell-surface ligands on T cells. These findings demonstrate the power of this method to validate the specificity of the ligand and receptor interaction.

Another unique application of this method would be to study the autocrine effects of the ligand- receptor interaction at the single-cell level. The current methods for detecting ligand-receptor interactions for studies of gene expression or signaling are limited to large populations of cells. Moreover, the impact of signaling on ligand-receptor interactions could be due to direct autocrine effects or to more indirect effects. The method described in this study uses the ligand to capture a single target cell, which can be subsequently stained for expression of a gene of interest (either known or unknown gene expression downstream of the ligand-receptor interaction). As such, researchers could conceivably use this technique to validate known pathways or reveal novel signaling pathways at a single-cell level. Additionally, as the distribution of cells on the slide is fairly uniform, it would be possible to use a hydrophobic marker to the divide the slide into several sections and stain for a panel of different genes using microscopy. This application may yield insights in mechanistic studies. Alternatively, imaging mass cytometry may be used in place of fluorescent staining, as this allows a greater number of targets to be identified.

## Conclusions

This protocol was originally developed to capture cell-surface vimentin (CSV)-positive circulating tumor cells (CTCs) using conventional antibody-antigen interaction mechanics. In the first iteration, CTCs flowed through the chip at a low rate and bound to an anti-CSV antibody coating on the slide chip. In the current study, we set out to develop a microfluidics test that demonstrated the preferential binding of wild-type (WT) T cells to FGL2 in comparison to FcγRIIB-knockout (KO) T cells. Our previous research showed that T cells express FGL2 receptors that interact with FGL2 ligands on glioma cells [9].

To demonstrate differences in ligand-receptor binding preferences, we repurposed the Abnova CytoQuest Circulating Rare Cell Positive Enrichment and Retrieval System for agnostic use. The CytoQuest system was developed to isolate CTCs based on the expression of CSV. Our lab replaced the anti-CSV antibody with the FGL2 protein to ensure capture of WT T cells. We also tested this protocol on cells expressing a membrane-anchored and tumor-targeted IL-12 (ATTIL-12) and ATTIL-12 KO cells to demonstrate that our platform is open ended and ligand/receptor agnostic.

A key feature of our method is its automation. As such, it is a user-friendly process that does not require highly trained personnel. The automation of the wash steps ensures that only cells that do not adhere to the chip are washed away. Another key feature of this method is its visualization facet, which allows investigators to directly observe binding activity. Inclusion of internal negative controls (FcγRIIB-KO T cells) allows definitive confirmation of binding based on visualization.

## Supporting information

S1 File (PDF)

## References

[1] Levi-Schaffer F. & Mandelboim O. Inhibitory and Coactivating Receptors Recognising the Same Ligand: Immune Homeostasis Exploited by Pathogens and Tumours. *Trends Immunol*. 39, 112–122 (2018). doi: 10.1016/j.it.2017.10.001 29066058 PMC7106362

[2] Zhao M., Jung Y., Jiang Z. & Svensson K. J. Regulation of Energy Metabolism by Receptor Tyrosine Kinase Ligands. *Front*. *Physiol*. 11, (2020). doi: 10.3389/fphys.2020.00354 32372975 PMC7186430

[3] Rho J. M. & Storey T. W. Molecular ontogeny of major neurotransmitter receptor systems in the mammalian central nervous system: Norepinephrine, dopamine, serotonin, acetylcholine, and glycine. *J*. *Child Neurol*. 16, 271–280 (2001). doi: 10.1177/088307380101600407 11332462

[4] Van Oss C. J. Hydrophobic, hydrophilic and other interactions in epitope-paratope binding. *Mol*. *Immunol*. 32, 199–211 (1995). doi: 10.1016/0161-5890(94)00124-j 7534869

[5] De Wildt R. M. T., Tomlinson I. M., Ong J. L. & Holliger P. Isolation of receptor-ligand pairs by capture of long-lived multivalent interaction complexes. *Proc*. *Natl*. *Acad*. *Sci*. *U*. *S*. *A*. 99, 8530–8535 (2002). doi: 10.1073/pnas.132008499 12084913 PMC124300

[6] De Jong L. A. A., Uges D. R. A., Franke J. P. & Bischoff R. Receptor-ligand binding assays: Technologies and applications. *J*. *Chromatogr*. *B Anal*. *Technol*. *Biomed*. *Life Sci*. 829, 1–25 (2005). doi: 10.1016/j.jchromb.2005.10.002 16253574

[7] Zeng S., Baillargeat D., Ho H. P. & Yong K. T. Nanomaterials enhanced surface plasmon resonance for biological and chemical sensing applications. *Chem*. *Soc*. *Rev*. 43, 3426–3452 (2014). doi: 10.1039/c3cs60479a 24549396

[8] Ogura T., Tanaka Y. & Toyoda H. Whole cell-based surface plasmon resonance measurement to assess binding of anti-TNF agents to transmembrane target. *Anal*. *Biochem*. 508, 73–77 (2016). doi: 10.1016/j.ab.2016.06.021 27349512

[9] Yan J., et al. FGL2 promotes tumor progression in the CNS by suppressing CD103+ dendritic cell differentiation. *Nat*. *Commun*. 10, (2019). 10.1038/s41467-018-08271-x PMC6347641 30683885

[10] Denisenko E., et al. Systematic assessment of tissue dissociation and storage biases in single-cell and single-nucleus RNA-seq workflows. *Genome Biol*. 21, (2020). doi: 10.1186/s13059-020-02048-6 32487174 PMC7265231

[11] Heinz S. & Glass C. K. Roles of lineage-determining transcription factors in establishing open chromatin: Lessons from high-throughput studies. *Curr*. *Top*. *Microbiol*. *Immunol*. 356, 1–15 (2012). doi: 10.1007/82_2011_142 21744305

[12] Zborowski M. & Chalmers J. J. Rare cell separation and analysis by magnetic sorting. *Anal*. *Chem*. 83, 8050–8056 (2011). doi: 10.1021/ac200550d 21812408 PMC3205221

[13] Basu S., Campbell H. M., Dittel B. N. & Ray A. Purification of specific cell population by fluorescence activated cell sorting (FACS). *J*. *Vis*. *Exp*. (2010) doi: 10.3791/1546 20644514 PMC3144656

[14] Pierce M. M., Raman C. S. & Nall B. T. Isothermal titration calorimetry of protein-protein interactions. *Methods A Companion to Methods Enzymol*. 19, 213–221 (1999). doi: 10.1006/meth.1999.0852 10527727

[15] Chi E. Y., Krishnan S., Randolph T. W. & Carpenter J. F. Physical stability of proteins in aqueous solution: Mechanism and driving forces in nonnative protein aggregation. *Pharm*. *Res*. 20, 1325–1336 (2003). doi: 10.1023/a:1025771421906 14567625

[16] Dobson C. M. Principles of protein folding, misfolding and aggregation. *Semin*. *Cell Dev*. *Biol*. 15, 3–16 (2004). doi: 10.1016/j.semcdb.2003.12.008 15036202

[17] Draczkowski P., Matosiuk D. & Jozwiak K. Isothermal titration calorimetry in membrane protein research. *J*. *Pharm*. *Biomed*. *Anal*. 87, 313–325 (2014). doi: 10.1016/j.jpba.2013.09.003 24119484

[18] Yakimchuk K. Protein Receptor-Ligand Interaction/Binding Assays. *Mater*. *Methods* 1, (2011).

[19] Nguyen H. H., Park J., Kang S. & Kim M. Surface plasmon resonance: A versatile technique for biosensor applications. *Sensors (Switzerland)* 15, 10481–10510 (2015). doi: 10.3390/s150510481 25951336 PMC4481982

[20] Liu H., et al. The FGL2-FcγRIIB pathway: A novel mechanism leading to immunosuppression. *Eur*. *J*. *Immunol*. 38, 3114–3126 (2008). 18991288 10.1002/eji.200838338

