# Supplementary material for: A microfluidics-based method for isolation and visualization of cells based on receptor-ligand interactions
Source: PLoS One. 2022 Oct 6;17(10):e0274601. doi: 10.1371/journal.pone.0274601 (PMC9536614; doi:10.1371/journal.pone.0274601)
Supplement: S1 File — (PDF) [file pone.0274601.s001.pdf]

Receptor-Ligand Visualization

Long Dao[1]

[1]University of Texas MD Anderson Cancer Center

Sep 06, 2022

1 Works for me Share

dx.doi.org/10.17504/protocols.io.e6nvwjp32lmk/v1

Long Dao

ABSTRACT

Receptor-ligand binding has been analyzed at the protein level using isothermal titration calorimetry and surface plasmon resonance and at the cellular level using interaction-associated downstream gene induction/suppression. However, no currently available technique can characterize this interaction directly through visualization. In addition, all available assays require a large pool of cells; no assay capable of analyzing receptor-ligand interactions at the single-cell level is publicly available. Here, we describe a new microfluidic chip–based technique for analyzing and visualizing these interactions at the single-cell level. First, a protein is immobilized on a glass slide and a low-flow-rate pump is used to isolate cells that express receptors that bind to the immobilized ligand. Specifically, we demonstrate the efficacy of this technique by immobilizing biotin-conjugated FGL2 on an avidin-coated slide chip and passing a mixture of GFP-labeled wild-type T cells and RFP-labeled FcγRIIB-knockout T cells through the chip. Using automated scanning and counting, we found a large number of GFP+ T cells with binding activity but significantly fewer RFP+ FcγRIIB-knockout T cells. We further isolated T cells expressing a membrane-anchored, tumor-targeted IL-12 based on the receptor's affinity to vimentin to confirm the versatility of our technique. This protocol allows researchers to isolate receptor-expressing cells in about 4 hours for further downstream processing.

DOI

dx.doi.org/10.17504/protocols.io.e6nvwjp32lmk/v1

PROTOCOL CITATION

Long Dao 2022. Receptor-Ligand Visualization. **protocols.io**
https://protocols.io/view/receptor-ligand-visualization-cf9etr3e

Citation: Long Dao Receptor-Ligand Visualization https://dx.doi.org/10.17504/protocols.io.e6nvwjp32lmk/v1

This is an open access protocol distributed under the terms of the **Creative Commons Attribution License** (https://creativecommons.org/licenses/by/4.0/), which permits unrestricted use, distribution, and reproduction in any medium, provided the original author and source are credited

FUNDERS ACKNOWLEDGEMENT

Long Dao
Qingnan Zhao
Jiemiao Hu
Xueqing Xia
Qing Yang
Shulin Li

LICENSE

This is an open access protocol distributed under the terms of the Creative Commons Attribution License, which permits unrestricted use, distribution, and reproduction in any medium, provided the original author and source are credited

CREATED

Sep 06, 2022

LAST MODIFIED

Sep 06, 2022

PROTOCOL INTEGER ID

69638

Generation and labeling of cells

1 Extract and homogenize the spleens of FcγRIIB-KO (Taconic, model no. 580) and C57/BL6 mice (Jackson Labs, cat. no. 000664) with a 40-µm mesh filter.

2 Isolate T Cells from the homogenized cells by using a MojoSort Mouse CD3 T cell Isolation Kit (BioLegend, cat. no. 480023).

3 Label T cells from WT mice with CellTracker Green BODIPY dye (Thermo Fisher Scientific, cat. no. C2102), and T cells from FcγRIIB-KO mice with CellTracker Red CMTPX dye (Thermo Fisher Scientific, cat. no. C34552).

Immobilization of target protein

4 Immobilize FGL2 on the microfluidic chip by covering a polydimethylsiloxane-coated microfluidic chip with 1 mg/mL streptavidin (Sigma, cat. no. 189730) for 1 hour at room

Citation: Long Dao Receptor-Ligand Visualization https://dx.doi.org/10.17504/protocols.io.e6nvwjp32lmk/v1

This is an open access protocol distributed under the terms of the **Creative Commons Attribution License** (https://creativecommons.org/licenses/by/4.0/), which permits unrestricted use, distribution, and reproduction in any medium, provided the original author and source are credited

temperature.

5 Coat the chip with 32 µg of biotin-conjugated FGL2 suspended in 100 µL of phosphate-buffered saline (PBS) for 1 hour at room temperature.

Isolation

6 Mix 5 × 10^6 FcγRIIB-KO T cells (red) and 1.5 × 10^6 WT T cells (green) and resuspend in 100 µL of T Cell media. 2.

7 Connect a spiral chamber containing microfluidic tubing to the Abnova CytoQuest microfluidic pump.

8 Prime for capture of WT T cells by washing with PBS and water.

9 Attach the chamber to the pump and allow flow process to initiate.

10 Temporarily disconnect the chamber from the pump and place the end tubing into an Eppendorf tube containing the T-cell mixture. The pump will draw the T cells into the chamber tubing and then passed the cell mixture through the slide chip. Only cells that display strong binding to the recombinant FGL2 protein will remain on the microfluidic chip. Cells that do not bind strongly will pass through the microfluidic chip into a waste container.

Visualization

11 Image the microfluidic chip with an automated Keyence microscope (www.keyence.com, model no. BZX-710) at 20× magnification.

12 Assign individual set points and image for fluorescein isothiocyanate and phycoerythrin channels.

Enumeration

13 Use the BZX-800 analysis software (Keyence) to count the cells on the resulting images. Count individual images were 'stitch mode' in groups of 400 until every image is counted.

Citation: Long Dao Receptor-Ligand Visualization https://dx.doi.org/10.17504/protocols.io.e6nvwjp32lmk/v1

This is an open access protocol distributed under the terms of the **Creative Commons Attribution License** (https://creativecommons.org/licenses/by/4.0/), which permits unrestricted use, distribution, and reproduction in any medium, provided the original author and source are credited

Citation: Long Dao Receptor-Ligand Visualization https://dx.doi.org/10.17504/protocols.io.e6nvwjp32lmk/v1

This is an open access protocol distributed under the terms of the **Creative Commons Attribution License** (https://creativecommons.org/licenses/by/4.0/), which permits unrestricted use, distribution, and reproduction in any medium, provided the original author and source are credited
